# Stereotactic radiosurgery may contribute to overall survival for patients with recurrent head and neck carcinoma

**DOI:** 10.1186/1748-717X-5-51

**Published:** 2010-06-09

**Authors:** Koji Kawaguchi, Kengo Sato, Akihisa Horie, Susumu Iketani, Hiroyuki Yamada, Yasunori Nakatani, Junichi Sato, Yoshiki Hamada

**Affiliations:** 1 Department of Oral and Maxillofacial Surgery, Tsurumi University, School of Dental Medicine. 2-1-3 Tsurumi, Tsurumi-ku, Yokohama, 230-8501, Japan; 2 Yokohama CyberKnife Center 574-1 Ishizawacyo, Asahi-ku, Yokohama, 241-0014, Japan; 3 Department of Oral and Maxillofacial Surgery, Toshiba Rinkan Hospital 7-9-1 Kamitsuruma, Sagamihara, 228-0802, Japan

## Abstract

**Background:**

The aim of this study is to examine the effect of stereotactic radiosurgery (SRS) in the treatment of advanced, recurrent lesions for head and neck carcinoma both with and without lymph node involvement.

**Methods:**

Between April 2006 and July 2007, 22 patients (mean age 67 years) with advanced, recurrent head and neck carcinoma were treated with stereotactic radiosurgery. All of the patients except one had biopsy confirmed disease prior to stereotactic radiosurgery. Patients included 3 rT2, 8 rT3, and 9 rT4; 8 of the patients had lymph node metastases. Marginal SRS doses were 20-42 Gy delivered in two to five fractions. Starting one month after SRS, all patients received S-1 oral chemotherapy for one year.

**Results:**

At an overall median follow-up of 24 months (range, 4-39 months), for the 14 locally recurrent patients without lymph node metastases, 9 patients (64.3%) had a complete response (CR), 1 patient (7.1%) had a partial response (PR), 1 patient (7.1%) had stable disease (SD), and 3 patients (21.4%) had progressive disease (PD). For the 8 patients with lymph node metastases, 1 patient with a single retropharyngeal (12.5%) had CR; the remaining 7 patients (87.5%) all progressed. Nine patients have died from their cancer. The overall actuarial 2-year survival for the patients with and without lymph node metastases is 12.5% and 78.6%, respectively.

**Conclusions:**

These results show the benefit of stereotactic radiosurgery salvage treatment for advanced, recurrent lesions, without lymph node metastases in previously irradiated head and neck cancer.

## Background

The majority of head and neck region squamous cell carcinomas present at an advanced stage and are treated with a combined-modality approach that often includes surgery, radiation therapy, and chemotherapy. Despite such aggressive approaches, advanced squamous cell carcinoma of the head and neck tends to recur locoregionally and, thus, presents a significant clinical challenge [1-3]. Surgery and/or conventional chemoradiotherapy salvage is difficult in advanced, recurrent lesions of head and neck carcinoma given the proximity of critical organs. Similarly, radical treatment for wide recurrent lesions is limited by overall radiation doses for the body and the possibility of severe post-operative dysfunction. In cases where further surgery is not feasible, reirradiation offers the potential to gain locoregional control and achieve palliation [4].

Several studies have confirmed the feasibility of reirradiation of recurrent head and neck tumors, with curative intent using external beam radiation therapy (EBRT) [5-8]. More recently, stereotactic radiosurgery (SRS) has been employed in the treatment of head and neck cancers both in primary cases [9,10] and in recurrent cases [11-15]. The CyberKnife^® ^(Accuray Incorporated, Sunnyvale, California, USA) is a frameless robotic radiosurgery system that has been utilized by numerous clinicians around the world to treat intracranial and extracranial tumors [9,13,16,17]. The CyberKnife image-guided radiosurgical system can deliver isocentric or non-isocentric beams with high precision and high dose conformity [18]. These abilities are especially important when treating irregularly shaped tumors, or those in difficult locations close to critical structures, as is the case with many of the patients with advanced, recurrent head and neck cancer. Here, we report on the tumor response and overall survival of stereotactic radiosurgery treatment using the CyberKnife for advanced, recurrent head and neck carcinoma lesions both with and without lymph node metastases.

## Methods

Between April 2006 and July 2007, 22 patients with advanced, recurrent head and neck squamous cell carcinoma were treated at the Yokohama CyberKnife Center, Yokohama, Japan. All patients included in this study completed an informed consent form. Patients were examined using PET-CT, MRI, and Ultrasound. Prior to radiation therapy, biopsies were performed for all patients, except for one for whom the recurrence was located deep in their temple muscle. For that patient, diagnosis was determined based upon the high level of uptake observed on PET scan. We assumed that this was a metastasis from the original squamous cell carcinoma that was located on the floor of their oral cavity. Hence, we denoted this patient as M1.

Prior to stereotactic radiosurgery, 21 patients (95.5%) had surgical treatment and 13 of those patients received post-operative chemo-radiotherapy. The remaining patient (4.7%) received prior irradiation, but neither prior surgery nor chemotherapy. The prior conventional irradiation was delivered as a wide field with doses of 40-65 Gy delivered in 1.5-2.0 Gy daily fractions. In all cases, the SRS was delivered within the prior irradiation field. The overall interval between prior treatment and SRS treatment was a median 11 months (range, 4-21 months). For those patients that received prior irradiation, SRS treatment occurred a median 11 months (range, 4-21 months) later. For those that received surgery alone, SRS treatment occurred a median 14 months (range, 7-26 months) later. Table 1 provides an overview of the patient characteristics.

**Table 1:** Patient characteristics.

Sex	Number (%)
Male	8 (36%)

Female	14 (64%)

Age, years	

Median (range)	67 (42-91)

Initial treatment	

Surgery alone	8 (36%)

Surgery + Post-operative Chemo-Radiation	13 (59%)

Radiation	1 (5%)

Prior radiation dose (Gy)	

Total (daily) dose range	40-65 (1.5-2)

Histology	

Squamous cell carcinoma	21 (95.5%)

M1 case	1 (4.5%)

Regions of recurrence or metastases	

Tongue	7 (31.8%)

Mandible	5 (22.7%)

Maxilla	3 (13.6%)

Maxillary sinus	3 (13.6%)

Soft palate	2 (9.1%)

Limited with lymph node	1 (4.5%)

Temporal muscle	1 (4.5%)

Clinical Stage	

rT0N1M0	1 (4.5%)

rT0N0M1	1 (4.5%)

rT2N0M0	3 (13.6%)

rT3N0M0	5 (22.7%)

rT3N1M0	2 (9.1%)

rT3N2M0	1 (4.5%)

rT4N0M0	5 (22.7%)

rT4N1M0	2 (9.1%)

rT4N2M0	2 (9.1%)

Stereotactic radiosurgery was delivered with the CyberKnife^® ^(Accuray Inc., Sunnyvale, USA), an X-band linear accelerator with an overall system targeting error of less than 1 mm [18,19]. The lightweight linear accelerator is capable of irradiating the target from 120 different directions using image-guidance based on a treatment plan created using a CT volume [13,20,21]. To assist with treatment planning, the CT image was also fused with an MRI or PET-CT image as applicable. During both treatment planning and delivery, patients were imaged while wearing a custom-made mouthpiece, to immobilize the moving parts of the mouth, and a thermoplastic mask fixed to the treatment couch, to minimize head movement. Treatment was administered depending upon the configuration and volume of the tumor as determined by the treating radiation oncologist, neurosurgeon and oral and maxillofacial surgeon. Dose constraints were applied to nearby critical structures based upon the total dose and fractionation scheme. Specifically, the dose to brain stem, optic nerve, optic chiasm, retina, and spinal cord were each limited to 21-25 Gy; the dose to the carotid artery, esophagus, and larynx were each limited to 30-35 Gy; and the dose to the eye lens was limited to 7-10 Gy. The prescribed dose of radiation was administered to the clinical target volume without the addition of any margin, corresponding to the 80-85% isodose contour. In the cases of lymph nodes metastases, the lymph nodes were treated. In general, the dose to those lesions which previously received irradiation was reduced by 20% from that of those patients for which no prior radiation was delivered to the lesion. In those cases where the PTV was more than 30 cc the dose was reduced by 30%. Overall, patients were treated with a median marginal dose of 33.73 Gy (range, 20-42 Gy) in two to five fractions with treatment delivered over consecutive days. The median gross lesion diameter was 36.63 mm (range, 15.21-58.65 mm). The median irradiated volume was 24.5 cm^3 ^(range, 3.4-74.4 cm^3^). Table 2 provides a summary of the treatment details.

**Table 2:** Treatment Characteristics: Summary of treatment dose and treated tumor volume.

Dose (Gy)	Number of Patients (%)
20 - 29	1 (4.5%)

30 - 34	7 (31.8%)

35 - 39	10 (45.5%)

40 - 42	4 (18.2%)

Median (range)	33.7 (20-42)

**Tumor volume (cm^3^)**	

Median	24.5

Range	3.4 - 74.4

**Tumor diameter (mm)**	

Median	36.63

Range	15.21 - 58.65

In addition to stereotactic radiosurgery, a low dose of oral chemotherapy S-1 (oral 5-FU prodrug) (Taiho Pharmaceutical Company Limited, Tokyo, Japan) was administered to control micro-lymph node metastases and distant metastases. The S-1 treatment began one month after SRS and consisted of 40-80 mg/body of S-1 for 2 weeks followed by a one week break; the treatment sequence was repeated for one year.

Following stereotactic radiosurgery, patients were monitored at either Tsurumi University Hospital or Toshiba Rinkan Hospital by oral and maxillofacial surgeons and at Yokohama CyberKnife Center by radiation oncologists. The clinical follow-up interval was every 2 weeks for the first 3 months, and every 4 weeks thereafter until the patient reached 2 years follow-up. Treatment outcome was assessed based on the Response Evaluation Criteria in Solid Tumors (RECIST) [22]. Response to treatment was evaluated using MRI at one month follow-up, contrast-enhanced CT at two months follow-up, PET-CT and MRI at three months follow-up, and MRI or PET-CT every three months thereafter. Toxicities were graded using the National Cancer Institute Common Toxicity Criteria Scale, Version 3.0. Overall survival after stereotactic radiosurgery was determined by Kaplan-Meier survival analysis.

## Results

### Clinical Outcomes

Twenty-two advanced, recurrent head and neck cancer patients were treated with SRS. In these advanced, recurrent patients treatment options were limited and the decision to treat these patients with SRS was based upon a variety of issues including the nature of the tumor recurrence, prior treatment approaches and patient preference. Specifically, the recurrent tumors were solid masses that were well suited to a radiosurgical treatment given the proximity of critical organs. For those patients that previously received conventional radiotherapy (14/22), the ability to target the radiation dose specifically to the tumor and limit the dose to surrounding, previously irradiated, tissue was also a strong indicator for SRS. Lastly, the patients strongly preferred a treatment option that did not require hospitalization.

The majority of patients (12/22) received SRS treatment as outpatients with curative intent. Ten patients (45%), however, received treatment with palliative intent while in terminal care at the hospital. At an overall median follow-up of 24 months (range, 4-39 months), 9 patients have died from their cancer. One additional patient died from acute cardiac insufficiency. For surviving patients, the median follow-up was 32 months (range, 27-39 months).

Table 3A and Table 3B provide a summary of tumor response as assessed by RECIST criteria based on lymph node metastases and clinical stage, respectively. Specifically, for the 14 locally recurrent patients without lymph node metastases, 9 patients (64.3%) had a complete response (CR), 1 patient (7.1%) had a partial response (PR), 1 patient (7.1%) had stable disease (SD), and 3 patients (21.4%) had progressive disease (PD). The three PD patients developed new lymph node metastases on the side opposite of SRS treatment. All three of these PD patients subsequently died from these late-lymph node metastases. For the 8 patients with lymph node metastases, one 1 patient, with only 1 retropharyngeal lymph node metastasis (12.5%) had CR; the remaining 7 patients (87.5%) all progressed. These seven patients each had 2 or 3 lymph node metastases located in their necks; upon progression they did not undergo additional treatment.

**Table 3:** Clinical Outcomes: Summary of tumor response by RESIST criteria.

3A				
**N-patients**		**Number of Patients (%)**		

Complete Response		9 (64.3%)		

Partial Response		1 (7.1%)		

Stable Disease		1 (7.1%)		

Progressed Disease		3 (21.4%)		

**N+ patients**				

Complete Response		1 (12.5%)		

Progressed Disease		7 (87.5%)		

**3B**				

**Clinical Stage**	**Complete Response**	**Partial Response**	**Stable Disease**	**Progressed Disease**

rT0N1M0	1			

rT0N0M1			1	

rT2N0M0	3			

rT3N0M0	3	1		1

rT3N1M0				2

rT3N2M0				1

rT4N0M0	3			2

rT4N1M0				2

rT4N2M0				2

(A) Tumor response for patients without lymph node metastases (N-) and for patients with lymph node metastases (N+). (B) Tumor response based on clinical stage.

**Figure 1:**
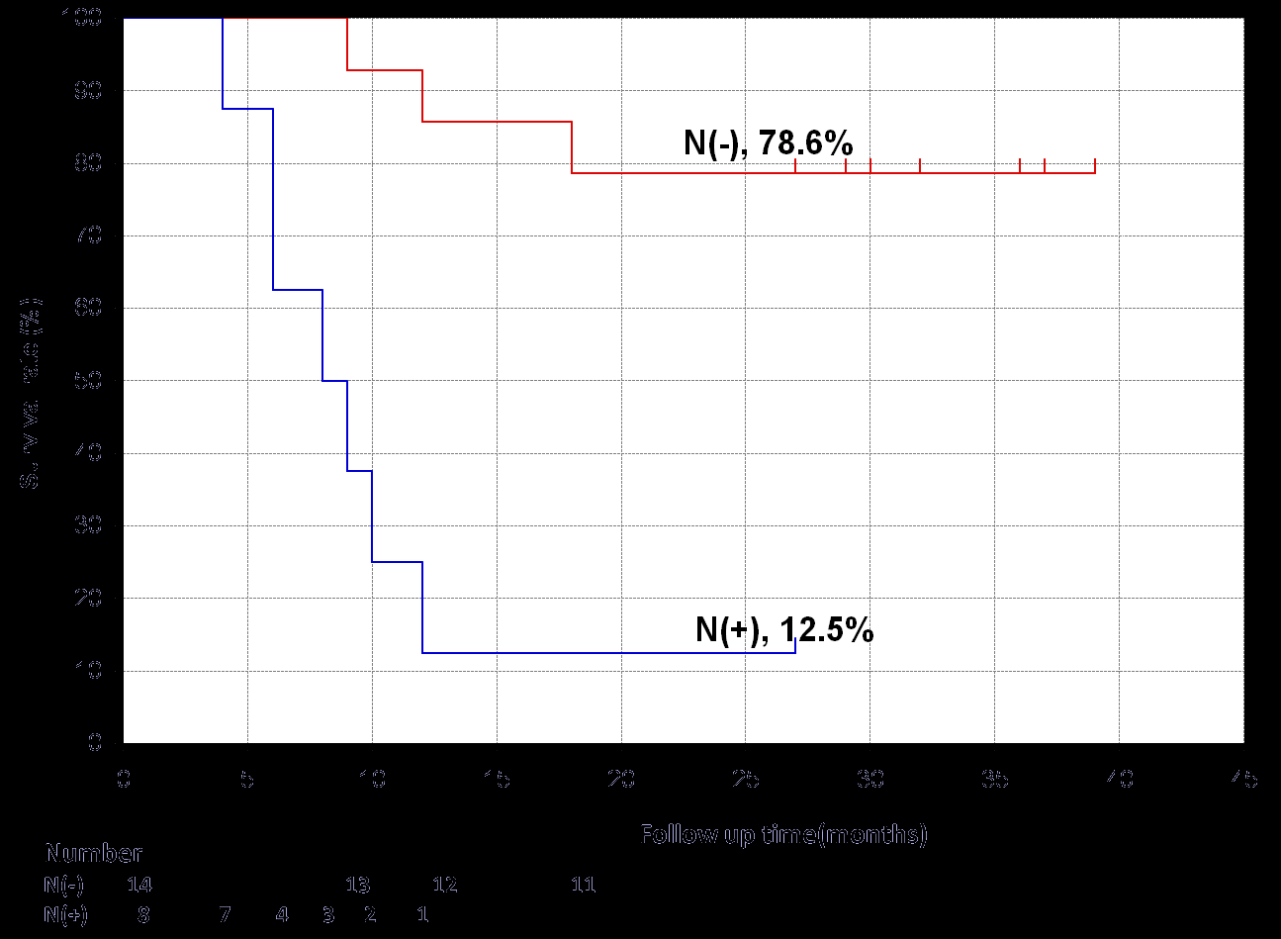
**Kaplan-Meier plot showing the overall survival rate for patients without lymph node metastases (N-) and for patients with lymph node metastases (N+)**.

Overall, at a median 2-years follow-up, 10 (45.5%) of the 22 severe recurrent cases maintained a complete response. All 10 of these patients have returned to society and regained quality of life. The overall actuarial 2-year survival for the locally recurrent patients with and without lymph node metastases is 12.5% and 78.6%, respectively (Figure 1). This difference was statistically significant with *p *= 0.000019 by the log-rank test.

### Complications

The first month following SRS 17 patients (77.3%) experienced Grade 2 xerostomia and decreased taste; 5 patients (22.7%), all of which were rT4 cases, experienced Grade 3 xerostomia and decreased taste. Of the 5 patients that experienced Grade 3 toxicity, one received prior radiation therapy (total dose 65 Gy) and four received prior surgery followed by radiation therapy (total dose 50-56 Gy). After the two month follow-up, there have been no serious complications associated with the SRS re-irradiation. Fourteen of the patients who had previously received external beam radiation experienced Grade 1 (11 patients) and Grade 2 (3 patients) osteoradionecrosis at 10-18 months after SRS. None of the surgery-only patients experienced any late complications.

### Case reports

**Figure 2:**
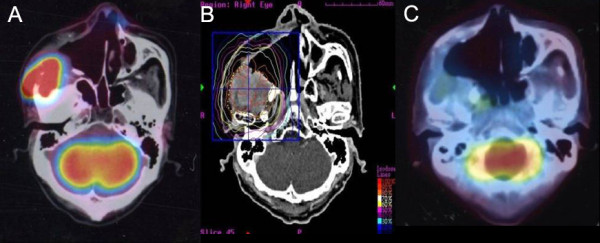
**Case study of a 59 year old patient with a locally recurrent lesion with pterygopalatine fossa 3-years after maxillectomy**. (A) Prior to treatment. (B) Treatment planning image. A total dose of 40 Gy was delivered in 5 fractions. (C) At 4-months post-treatment a complete response occurred.

**Case One (Figure **2**): **A 59-year-old male that was found to have a locally recurrent lesion with pterygopalatine fossa 3-years after maxillectomy. SRS was delivered to a total dose of 40 Gy in 5 fractions. The patient experienced Grade 2 xerostomia and decreased taste without osteoradionecrosis within the first month of treatment. Four months after SRS a complete clinical response occurred. At 30 months after SRS there is no evidence of recurrence.

**Figure 3:**
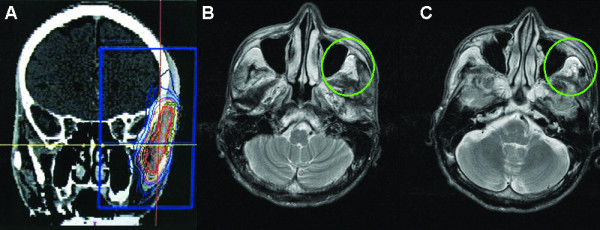
**Case study of a T3N2cM0 patient, with tongue carcinoma that recurred as a distant metastasis in part of his temporal muscle 5 years after surgery**. (A) Treatment planning image. A total dose of 30 Gy was delivered in 3 fractions. (B) Pre-treatment the recurrence is visible in his temporal muscle as denoted by the green circle. (C) At 3-months post-treatment the lesion was stable (green circle).

**Case Two (Figure **3**): **A patient with T3N2cM0 tongue carcinoma that recurred as a distant metastasis in part of his temporal muscle 5 years after surgery. SRS was delivered to a total dose of 30 Gy in 3 fractions. Three months after SRS the lesion was assessed as stable and remains as stable disease at 24 months. The patient experienced hair loss at the temporal part of his head within 3 months after SRS, after which the hair grew back.

**Figure 4:**
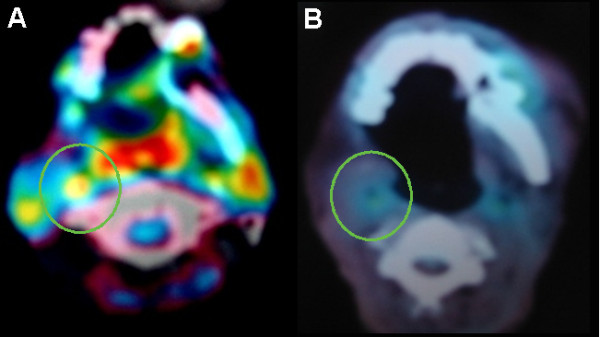
**Case study of a patient with T3N2cM0 tongue carcinoma that recurred as a retropharyngeal lymph node metastasis 5 years after surgery**. (A) Pre-treatment the lymph node metastasis is indicated by the green circle. (B) At 5-months post-treatment a complete response occurred (green circle).

**Case Three (Figure **4**): **A patient with T3N2cM0 tongue carcinoma that recurred with a retropharyngeal lymph node metastasis 5 years after surgery. SRS was delivered to a total dose of 23 Gy in 2 fractions. Three months after SRS the lesion exhibited a complete clinical response. At 26 months after SRS there is no evidence of recurrence. This patient was the only one of eight treated lymph node metastases in our study that had a complete response.

**Figure 5:**
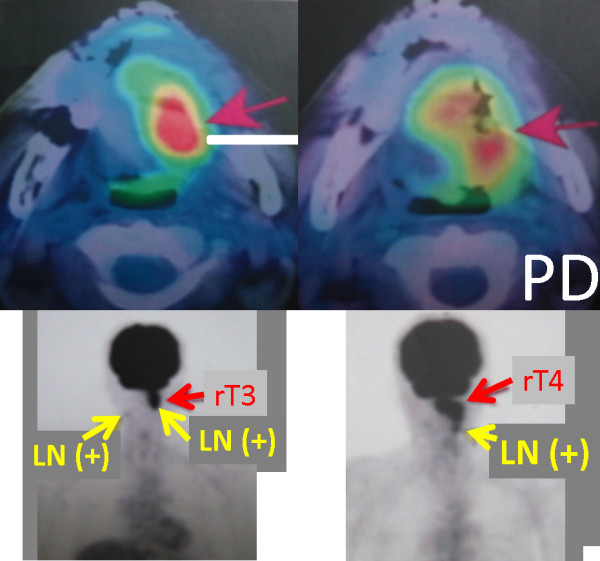
**Case study of a patient with rT3N2cM0 tongue carcinoma that recurred 6 months after irradiation with 50 Gy in 25 fractions by conventional external beam**.

**Case Four (Figure **5**): **A patient with rT3N2cM0 tongue carcinoma recurred 6 months after irradiated with 50 Gy in 25 fractions by conventional external beam. SRS was delivered to a total dose of 35 Gy in 5 fractions. Three months after SRS for the recurrent lesion of the tongue and N+ lesions, these lesions increased in size and were assessed as progressive disease. The patient experienced Grade 3 xerostomia and decreased taste as well as Grade 2 osteoradionecrosis of the mandible bone. This patient died six months after SRS as a result of a large number of lymph node metastases.

## Discussion

Our results demonstrate that CyberKnife frameless stereotactic radiosurgery for patients with recurrent head and neck carcinoma is feasible and safe in the setting of previous irradiation. In the case of local recurrence without lymph node metastases, 9 out of 14 (64.3%) patients had a complete response with a 2-year overall survival rate of 78.6%. Overall, 10 out of 22 (45.5%) of the advanced, recurrent patients maintain a complete response at a median 2-years follow-up.

Several stereotactic radiosurgery results, also known as fractionated stereotactic radiotherapy (fSRT), for re-irradiation of recurrent head and neck carcinomas have been reported [13-15,23-25]. The complete response rates for these studies vary from 8.6-54% with 2-year overall survival rates ranging from 14.3-41% and 1-year overall survival rates of 18-52.1% (see Table 4). In addition, one study has reported 2-year overall survival rates of 58% for reirradiation of recurrent head and neck cancer using IMRT. As the ranges of these outcomes suggest, the heterogeneity between these various studies is large. Various factors, including tumor stage, tumor volume, adequate irradiation dose, prior treatment, and anatomical site complexly, influenced these reported outcomes. In several of these studies [13,14,23] patients with lymph node metastases have been included, but the reported outcomes have not been divided between those patients with and without lymph node metastases. Our reported overall survival of 78.8% for our subset of patients without lymph node involvement exceeds all of the prior published survival rates.

**Table 4:** Overview of prior stereotactic radiosurgery results for the reirradiation of recurrent head and neck carcinoma.

Study	Patients (#)	SRS median total dose/fx	Follow-up median, range (months)	Tumor size median, range (cc)	Prior irradiation Dose, median, range (Gy)	Toxicity	Complete Response Rate	Overall survival
Voynov et al. [13]	22	24/1-8	19, 11-40*	19.1, 2.5-140.3	97.8, 70.1-190.3 (BED_10_)	No Grade 4+	-	22% at 2-yrs

Heron et al. [15]	25	25-44/5	NS	44.8, 4.2-217	66-69.2	No Grade 3+	8.6%	18% at 1-yr

Roh et al. [14]	36	30/3-5	17.3	22.6, 0.2-114.9	70.2, 39.6-134.4	No Grade 4+ 36% Grade 3	42.9%	52.1% at 1-yr, 30.9% at 2-yrs

Rwigema et al. [23]	85	35/1-5	6, 1.3-39	25.1, 2.5-162	70, 32-170.7	No Grade 4+ 4.7% Grade 3	34%	48% at 1-yr, 16% at 2-yrs

Sulman et al. [31]	78^§^	IMRT 60 Gy	25, 0-81	64.1, 2.9-425.4	60, 16-75	20% severe Including 1% Grade 5	-	58% at 2-yrs

Siddiqui et al. [25]	44^§^†	13-18/1 or 36-48/5-8	6.8, 1.5-48	15.5, 1.7-155‡	63.5, 50.4-74‡	6.7% Grade 3‡ 9% Grade 4‡	31%	38.1% at 1-yr 14.3% at 2-yrs

Unger et al. [24]	65	30/2-5	16*	75, 7-276	67, 32-120	11% Grade 4+	54%	41% at 2-yrs

Current Study	22 (14 N-, 8 N+)	33.7/2-5	24, 4-39	24.5, 3.4-74.4	40-65	No Grade 4+ 22.7% Grade 3	45.4% (N- 64.3%, N+ 12.5%)	N- 78.6% at 2-yrs N+ 12.5% at 2-yrs

* Surviving patients

† Additional non-recurrent patients treated, data presented for recurrent patients only unless otherwise noted.

‡ Includes non-recurrent patients.

§ Includes patients with non-squamous cell carcinomas; data presented is for combined population.

Another factor affecting the reported overall survival rates is the use of chemotherapy. Recent evidence suggests that concurrent administration of chemotherapy may reduce the risk of micrometastases. A study of nasopharyngeal carcinoma demonstrated that progression-free survival among the patients who were treated with radiation alone was 24% at 3 years, compared to 69% in the combined treatment group [26]. In our study, starting one month post-SRS and continuing for one year, 100% (22/22) of the patients received low dose S-1 oral chemotherapy to maintain local control and to avoid lymph node and distant metastases. We choose to use S-1, an oral 5-fluorouracil (5-FU), based on several studies showing promising safety and efficacy results with this chemotherapy agent for the treatment of advanced head and neck squamous cell carcinoma [27-30]. This addition of S-1 to the SRS treatment may have also contributed to the satisfactory outcomes observed in this study.

While our results are very promising for cases without lymph node metastases, in the cases with lymph node metastases only 1 of 8 patients had a complete response and the 2-year overall survival rate was 12.5%. Since these lymph node metastases were mostly locally advanced lesions, neck dissection was not available. Given the low observed control rate, we recommend that patients eligible for surgery under general anesthesia undergo a combined salvage treatment strategy of neck dissection for the regional lymph node metastases and SRS for the locally recurrent lesion.

Reported toxicity rates for reirradiation of recurrent head and neck carcinoma include late Grade 4 and higher toxicity rates of 9% [25] and 11% [24], a rate of 36% Grade 3 toxicity [14], and a rate of 20% severe toxicity including 1% Grade 5 toxicity for an IMRT reirradiation study [31]. In comparison, our 22.7% rate of Grade 3 toxicity with no higher grade toxicities is promising.

## Conclusions

At a median 2-years follow-up, 45.5% (10/22) of the advanced recurrent patients maintained a complete response. For the local recurrent patients with non-lymph node metastases 64.3% (9/14) of patients had a complete response and the 2-year actuarial overall survival rate is 78.6%. Toxicity was acceptable with no observed grade 4 or higher toxicity. Hypofractionated robotic stereotactic CyberKnife radiosurgery treatment is feasible, safe, and well-tolerated for patients with local recurrence in head and neck carcinoma.

## Conflict of interests statement

The authors declare that they have no competing interests.

## Authors' contributions

KK and KS were responsible for the treatment of the patients and collection of data. All authors were responsible for gathering and interpreting data, manuscript revision and final manuscript approval.
